# Development of Aortic Stents from Open Surgery to Endovascular Therapy: A Brazilian Contribution to the Treatment of Aortic Diseases

**DOI:** 10.21470/1678-9741-2026-0082

**Published:** 2026-11-01

**Authors:** José Honório de Almeida Palma da Fonseca, Adnaldo da Silveira Maia, Gabriel Damasio Palma da Fonseca, Bruno Silva Ribeiro, Antônio Agostinho Moura Filho

**Affiliations:** 1 Cardiovascular Surgery Department, Instituto do Coração do Hospital das Clínicas da Faculdade de Medicina da Universidade de São Paulo (InCor/FMUSP), São Paulo, São Paulo, Brazil; 2 Department of Cardiovascular Surgery, Pontifícia Universidade Católica de Campinas (PUC-Campinas), Campinas, São Paulo, Brazil

**Keywords:** Aorta, Stents, Vascular Prostheses, Endovascular Surgery, History of Medicine.

## Abstract

The treatment of aortic diseases has undergone profound transformation over the last decades, with the progressive incorporation of endovascular techniques either replacing or complementing conventional open surgical repair. The development of aortic stents represents one of the most significant milestones in this transition. This article reviews the historical, experimental, and clinical evolution of aortic stents, with emphasis on pioneering Brazilian contributions, including the experimental phase in animal models, the first clinical applications in aortic dissection, and the role of the national industry in the development and dissemination of these technologies. The impact of these innovations on contemporary cardiovascular surgical practice and future perspectives are also discussed.

## INTRODUCTION

**Table:** 

Abbreviations, Acronyms & Symbols	
R	= Renal artery
SMA	= Superior mesenteric artery

For much of the 20^th^ century, the surgical treatment of aortic diseases was associated with extensive, highly invasive, and technically complex procedures, often performed under cardiopulmonary bypass, deep hypothermia, and circulatory arrest. Although effective, these approaches were associated with significant morbidity and mortality and important limitations, particularly in elderly patients or those with multiple comorbidities^[1]^.

The introduction of endovascular therapy represented a paradigm shift by proposing the treatment of aortic diseases through an intraluminal approach, with reduced surgical trauma, faster recovery, and greater technical reproducibility. In this context, aortic stents became the central element of this therapeutic revolution.

The possibility of endovascular treatment for arterial diseases was originally proposed by Charles Dotter in 1969. Subsequently, in 1991, Parodi et al. successfully demonstrated and disseminated the treatment of abdominal aortic aneurysms using balloon-expandable prostheses. In 1994, the Stanford University group published their initial experience in the treatment of descending thoracic aortic aneurysms in 13 patients, using self-expanding prostheses based on the same principle^[1-3]^.

## CONCEPTUAL AND EXPERIMENTAL PRINCIPLES OF AORTIC STENT DEVELOPMENT

The introduction of aortic stents in Brazil occurred in a scenario of intense technological innovation, but it was also marked by important limitations, such as restricted access to imported devices, which were themselves still under development, high costs, and the absence of solutions adapted to the national reality. In this context, Brazilian development assumed unique characteristics, based on the integration of experimental research, clinical practice, and local industry^[4]^.

In this process, the participation of the national industry, particularly Braile Biomédica (São José do Rio Preto, São Paulo, Brazil), was decisive. The company enabled the Brazilian development of aortic stents and endovascular grafts, allowing the transition from experimental concepts to clinical application. The direct collaboration between cardiovascular surgeons and biomedical engineers allowed rapid technical adjustments based on early clinical experience, facilitating the dissemination of endovascular therapy in the country and consolidating national technological autonomy in this field.

The application of the intravascular support concept to the aorta imposed specific challenges related to large vessel diameters, high pulsatile forces, and the anatomical complexity of the vessel. Unlike coronary and peripheral arteries, the aorta requires devices with high mechanical resistance, adequate conformability, and favorable biological interaction with the vascular wall^[4-9]^.

## BRAZILIAN PIONEERING EXPERIENCE AND NATIONAL DEVELOPMENT

The Brazilian experience in the development and clinical application of aortic endovascular grafts began in a pioneering manner in the late 1980s and early 1990s, at a time when endovascular technology was still in an embryonic stage on the international scene. During this period, systematic experiences with endovascular techniques applied to aortic diseases were initiated, involving experimental models, development of prosthetic devices, controlled delivery techniques, hybrid strategies, and even the use of endoscopic visualization of the aorta (Figure 1).

**Fig. 1:**
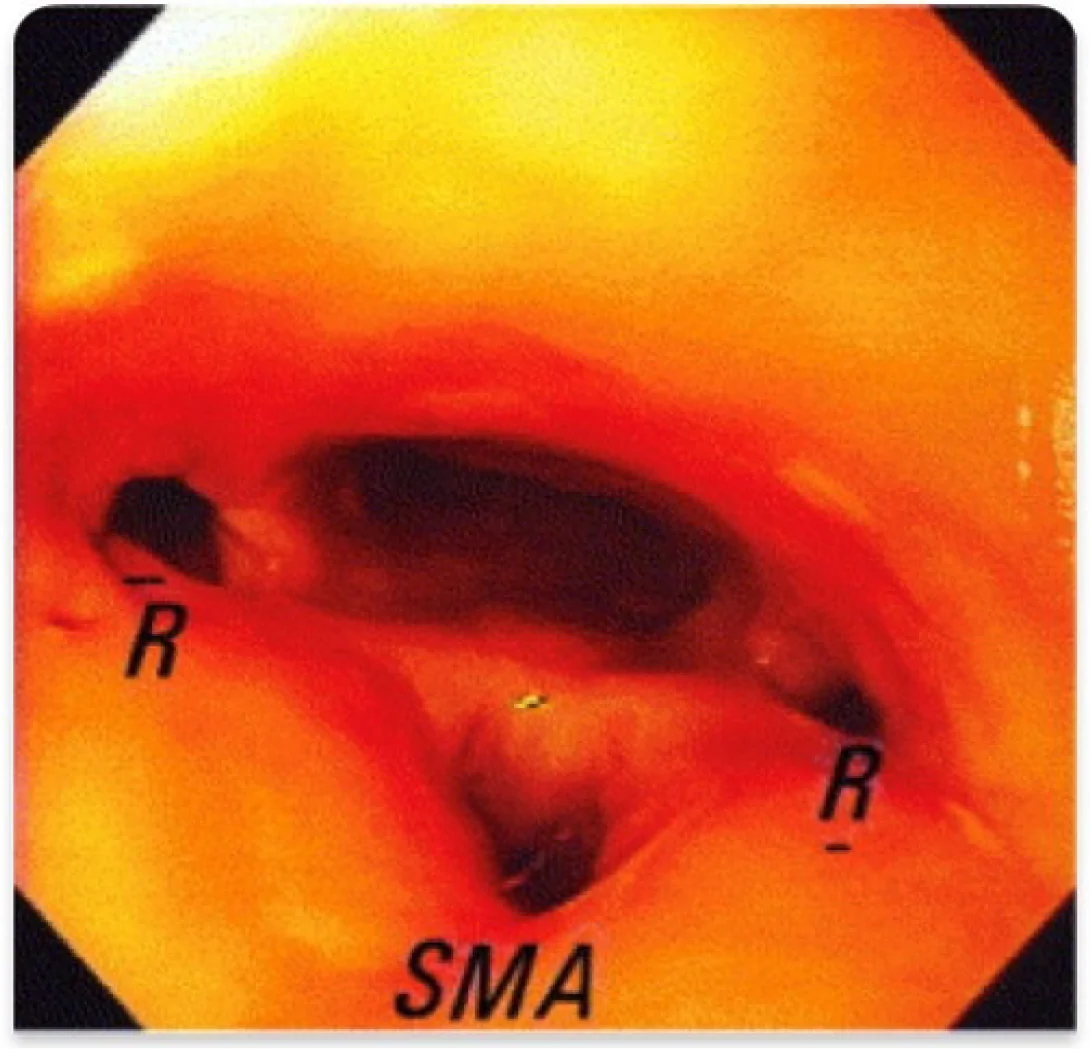
Endoscopic view of the interior of the abdominal aorta performed under total circulatory arrest, demonstrating the ostium of the right and left renal arteries and the ostium of the superior mesenteric artery. Adapted from Palma et al., Treatment of thoracic aortic aneurysms by the introduction of stents under endoscopic visualization; 1998^[4]^. R=renal artery; SMA=superior mesenteric artery.

The first Brazilian experiences with aortic stents were conducted in animal models within the Experimental Surgery Department of the Universidade Federal de São Paulo (or UNIFESP), aiming to evaluate technical feasibility, the biological response of the aortic wall, and the tissue integration process of endovascular grafts. Expandable metallic stents were surgically implanted in the aorta of animals with normal anatomy to evaluate the local biological response, device incorporation into the aortic wall, and the feasibility of the intraluminal concept (Figure 2).

**Fig. 2:**
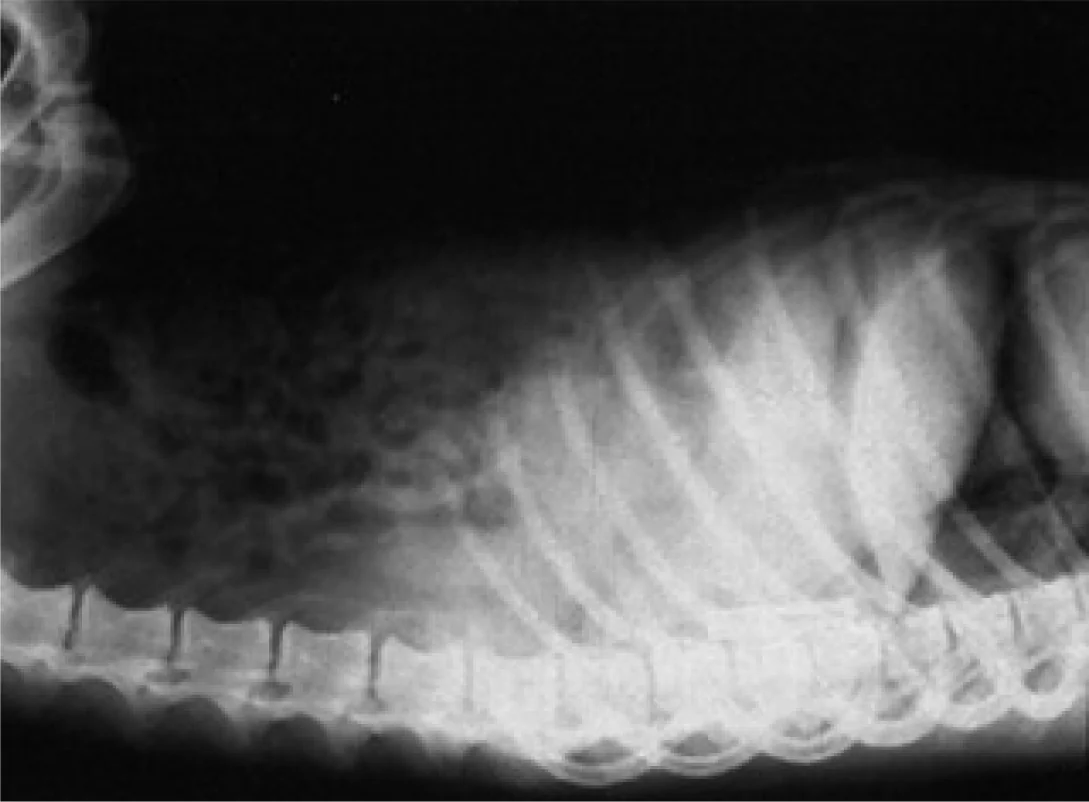
Endograft in the descending thoracic aorta in canine specimens. Adapted from Paula et al. Use of a self-expanding endoprosthesis stent positioned in the canine thoracic aorta. 1997^[5]^.

It was observed that as early as seven days after implantation, in certain portions of the aorta it was no longer possible to macroscopically identify the metallic structure of the stent, which was completely covered by neointimal tissue, demonstrating a rapid biological response to implantation (Figure 3). At 30 days, a continuous endothelial layer covering the lumen was observed, with the presence of fibrin and formation of new endothelium. At 60 days, the neointima was well established and associated with an underlying foreign body-type inflammatory reaction. At 180 days, complete integration of the prosthesis into the adjacent aortic wall was observed^[5]^.

**Fig. 3:**
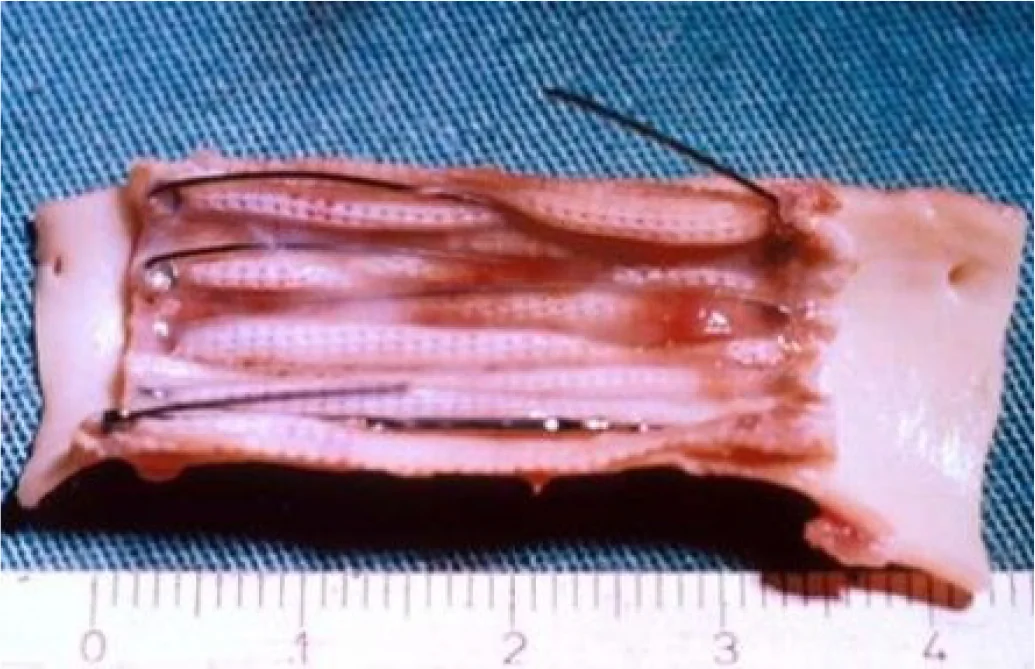
Internal view of the canine thoracic aorta with an incorporated stent. Adapted from Paula et al. Use of a self-expanding endoprosthesis stent positioned in the canine thoracic aorta. 1997^[5]^.

In parallel, the first national clinical studies were notable for the use of self-expanding endovascular grafts surgically implanted, often associated with the elephant trunk technique, with the aim of promoting aneurysm exclusion or stabilization of distal aortic dissection. These studies demonstrated the feasibility of introducing intraluminal prostheses into the aorta, anticipating concepts that would later be consolidated by modern hybrid and endovascular surgery^[9]^.

## INITIAL TREATMENT OF TYPE B AORTIC DISSECTION

Strongly influenced by the work of Professor Borst et al., who introduced the “Elephant Trunk” technique, our group innovatively and pioneeringly adapted this technique to treat type B aortic dissection. The main objective was to promote occlusion of the intimal tear located in the proximal descending thoracic aorta. This initiative represented a historical milestone by translating well-established open surgical concepts into a context that anticipated the foundations of modern endovascular therapy^[6-9]^.

With the progressive expansion of clinical experience and technological development of endovascular grafts, the technique was subsequently refined, allowing stent implantation in the descending thoracic aorta under direct visualization (Figure 4)^[9]^.

**Fig. 4:**
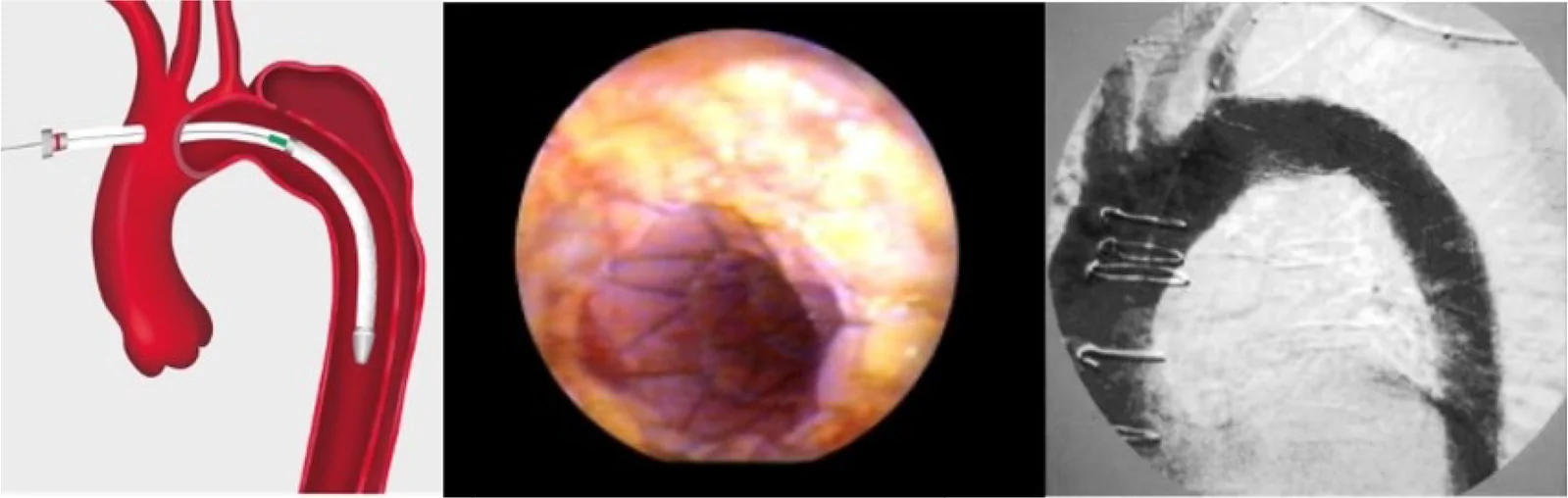
Catheter containing an aortic stent positioned in the descending thoracic aorta under direct visualization through the aortic arch. Stent released and positioned in the descending thoracic aorta. Final aspect visualized by arteriography. Source: Author’s archive.

It is noteworthy that, even before the first transfemoral implantation was performed, the group from the Escola Paulista de Medicina was already using aortic stents, in an unprecedented manner, in the treatment of type B aortic dissection. In this early phase, the devices were positioned through the aortic arch, excluding the entry tear and promoting restoration of predominant flow through the true lumen.

These procedures were performed in a surgical setting under deep hypothermia and total circulatory arrest, combining principles of open surgery with emerging concepts of endoluminal exclusion. At a time when the international literature did not recommend the use of aortic stents for the treatment of dissection, this hybrid strategy allowed understanding of device behavior in dissected aortas and anticipated fundamental concepts of modern endovascular treatment of type B aortic dissection.

Based on the favorable initial results obtained with the surgical insertion of stents through the aortic arch under direct visualization, fully endovascular implantation via femoral access was subsequently developed. From this experience, the progressive transition to exclusively endovascular treatment was consolidated through the percutaneous introduction of endovascular grafts, whose first implantation was performed in 1991 by Parodi et al. for abdominal aortic aneurysms^[2]^.

## FIRST TRANSFEMORAL ENDOVASCULAR IMPLANTATION IN DESCENDING AORTIC DISSECTION

The natural evolution of this experience culminated in the performance of the first transfemoral implantation of an aortic stent in a female patient with descending thoracic aortic dissection. At that time, this was an unprecedented procedure, performed in a context in which no author in the international literature advocated the use of aortic stents for this pathology^[10]^.

The therapeutic decision was based on the severity of the clinical condition, the absence of effective alternatives, and the knowledge accumulated from the experimental and hybrid phases. The procedure was successfully performed, demonstrating the technical feasibility of the endovascular approach in aortic dissections and representing a conceptual turning point that anticipated by several years the subsequent incorporation of this strategy into international guidelines (Figure 5)^[10,11]^.

**Fig. 5:**
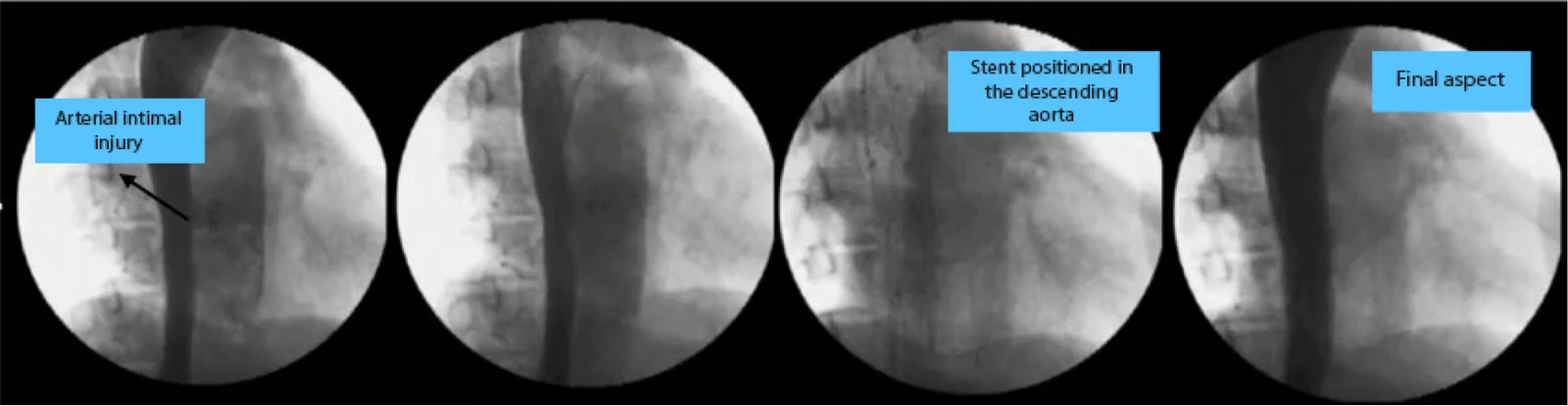
Demonstration of the intimal tear and false lumen in type B aortic dissection, positioning of the endovascular graft, and consequent exclusion of the false lumen. Adapted from Palma et al. Use of an expandable endoprosthesis (“stent”) introduced through the femoral artery for the treatment of descending aortic dissection. 1997.^[10,11]^.

## INSTITUTIONAL RECOGNITION AND SCIENTIFIC CONTRIBUTIONS

The impact and originality of this early phase were widely recognized within the national scientific community. The pioneering experiences developed by the group received formal recognition from the Sociedade Brasileira de Cirurgia Cardiovascular and the Sociedade Brasileira de Angiologia e Cirurgia Vascular, with the awarding of several prizes and honorable mentions at scientific congresses and events at the time. This recognition reflected the innovative nature of the proposed approaches and the strength of the experimental and clinical foundation that supported the introduction of aortic stents in Brazil.

## TECHNOLOGICAL EVOLUTION OF AORTIC STENTS

The evolution of aortic stents can be divided into different phases:

First generation: high-profile devices, rigid structures, and rudimentary delivery systems, associated with high complication rates.

Second generation: introduction of self-expanding nitinol stents, association with synthetic fabrics such as Dacron® and polytetrafluoroethylene, and improved adaptation to aortic curvatures.

Contemporary era: low-profile devices, precise delivery systems, fenestrated and branched endovascular grafts, as well as customized solutions for complex anatomies.

The incorporation of aortic stents has profoundly transformed the practice of cardiovascular surgery, reducing perioperative morbidity and mortality, expanding therapeutic indications, and promoting greater integration between surgeons and interventional specialists. The concept of the “Heart Team” has been consolidated, and the figure of the “hybrid surgeon”, proficient in both open surgery and endovascular techniques, has emerged^[12]^.

## CHALLENGES AND FUTURE DIRECTIONS

Despite significant advances in the endovascular treatment of aortic diseases, important challenges still persist, particularly those related to the long-term durability of devices, the fully endovascular treatment of the ascending aorta, and the development of more complex solutions, such as the concept of the “Endo-Bentall” (Figure 6), whose clinical application, although already performed, has been limited to highly selected cases^[13]^.

**Fig. 6:**
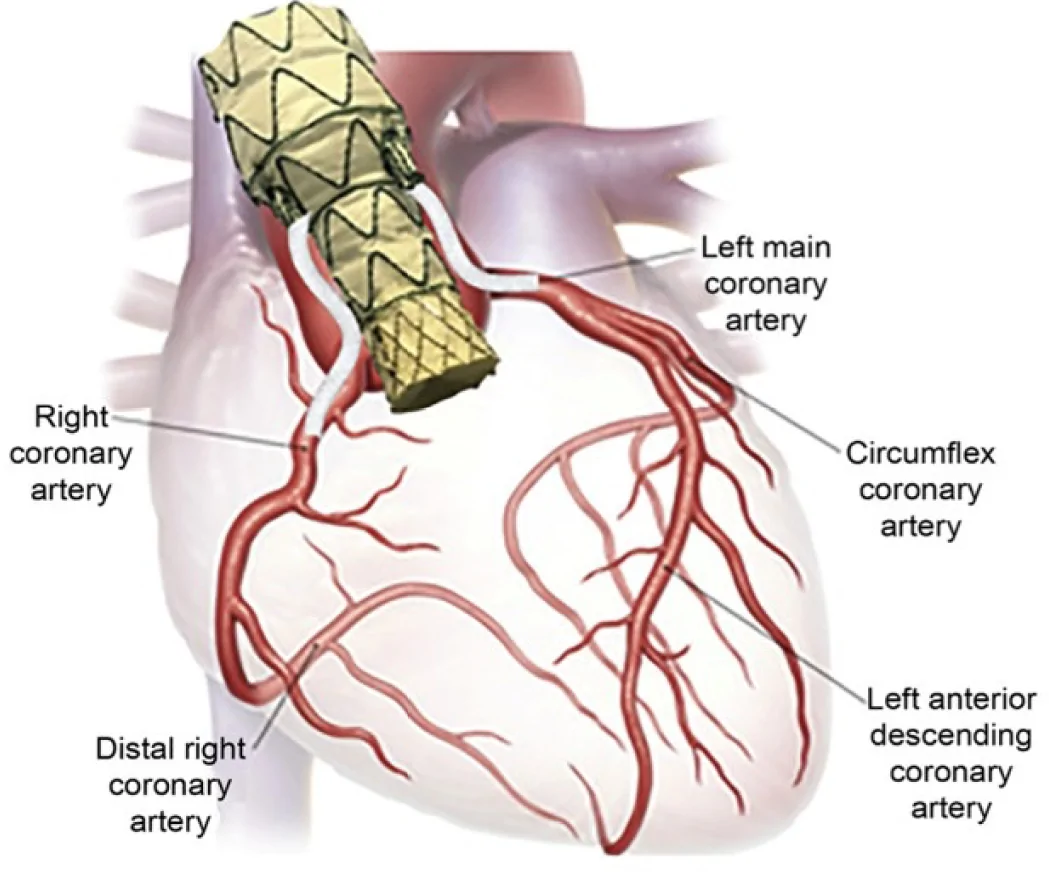
Illustration of the Endo-Bentall procedure. Adapted from Gaia et al. (2020)^[13]^.

Currently, there is increasing use of thoracic endovascular grafts in an off-label setting in the ascending aorta, especially in patients with aortic dissection and prohibitive surgical risk, in whom conventional surgery presents unacceptable morbidity and mortality. In parallel, endovascular grafts specifically designed for the aortic arch and ascending aorta are in different stages of development and clinical evaluation, representing a fundamental step toward consolidating a fully endovascular approach in these segments^[14,15]^.

In this context, the use of advanced planning based on computed tomography, combined with 3D printing and artificial intelligence tools, emerges as the next evolutionary stage of this technology, with the potential to optimize patient selection, personalize devices, increase implantation accuracy, and reduce complications, thereby safely expanding the indications for endovascular treatment of aortic diseases.

## CONCLUSION

The development of aortic stents represents one of the greatest revolutions in modern cardiovascular surgery. The Brazilian experience, initiated in the experimental laboratory, consolidated through hybrid approaches, and culminating in unprecedented clinical applications, has played a fundamental role in the evolution of this therapy. The integrated efforts of innovative surgeons, academic institutions, and the national industry have contributed decisively to the consolidation of endovascular therapy in Brazil and to its projection on the contemporary international stage.

## Data Availability

The authors declare that the data supporting the findings of this study are available within the article.

## Artificial Intelligence Usage

The authors declare that no artificial intelligence tool was used in the preparation of this article.
